# Autibiography of Chas. Caldwell, M. D.

**Published:** 1855-05

**Authors:** 


**Autibiography of Chas. Caldwell, M. D.**

To say that we were not interested in the perusal of this work, would not be a just expression of our feelings; and while we acknowledge that we were led on from page to page, culling in our progress a bright spot here, and something commendable there in the life and character of the author, we were pained to find so much to condemn and so much to deprecate in his career. From his own confessions he must have been a man of vanity, ambition, acerbity, vindictiveness and violence. These dominent feelings led him to assail the very best men of the profession of that day, because, it would appear, they would not, because they conscientiously could not favor his mad schemes of ambition and self-aggrandisement. The next step was disorganization, and this feature which makes itself manifest upon a perusal of the work, was an unfortunate one in his character.

Many years ago, after perusing his notes upon Cullen's work, we formed this opinion of the man. He attacked the work with such a ferocity, and so severely exposed its fallacies, that we thought he would consider it an easy task to furnish some better system instead. But although he proved competent to the work of destruction, he did not essay even to rebuild. To wipe out Cullen was to leave the ground unoccupied; to annihilate his opinions and doctrines, was but to leave a blank.

But if he was ambitious, and if indeed it proved a ruling passion with him, he was a man of talent and acquirement. Unlike many others, then and now, who were and are drunk with an ambition so powerful as to still all conscientous scruples of honor or principle, and are at the same time skimming along on a smooth surface but without a single substantial claim to talent; he was, however, a learned and accomplished physician. He had claims to prominence, because of his ability as a teacher and writer; but his vaulting ambition for still higher positions and power, overwhelmed him in difficulties and thwarted his designs. His was no modest merit, but so anxious was he for distinction, that he was not satisfied with a silent acknowledgement, but required an open public avowal of it. But amid all, he was a high-toned gentleman, whose aspirations were too lofty for the perpetration of a low, dishonest, or dishonorable act, however much it might result to his own benefit. His aim was high and his spirit lofty, if indeed he manifested too much of anxiety to attain to the mark and secure the prize.

The work should be read by every medical man, so that his virtues should be remembered to be imitated, and his follies avoided and forgotten. It is published by Lippincott, Gambo & Co., Philadelphia, and is neatly executed.

